# Supplementary material for: A comparative analysis of the COVID-19 Infodemic in English and Chinese: insights from social media textual data
Source: Front Public Health. 2023 Nov 10;11:1281259. doi: 10.3389/fpubh.2023.1281259 (PMC10686410; doi:10.3389/fpubh.2023.1281259)
Supplement: Supplementary file 1 [file Table_1.docx]

**Appendix 1:** Translation for Chinese displayed in Figure 1 and Figure 2

| All records | | Records labeled as true | | Records labeled as false | | Records labeled uncertain | |
| --- | --- | --- | --- | --- | --- | --- | --- |
| Chinese | Translation | Chinese | Translation | Chinese | Translation | Chinese | Translation |
| 病毒 | Virus | 病毒 | Virus | 病毒 | Virus | 肺炎 | Pneumonia |
| 肺炎 | Pneumonia | 口罩 | Mask | 肺炎 | Pneumonia | 武汉 | Wuhan |
| 口罩 | Mask | 肺炎 | Pneumonia | 口罩 | Mask | 病毒 | Virus |
| 疫情 | Epidemic | 患者 | Patient | 疫情 | Epidemic | 疫情 | Epidemic |
| 武汉 | Wuhan | 消毒剂 | Sanitizer | 患者 | Patient | 医院 | Hospital |
| 患者 | Patient | 症状 | Symptom | 美国 | U.S. | 美国 | U.S. |
| 美国 | U.S. | 医用 | Medical | 酒精 | Ethyl alcohol | 中国 | China |
| 钟南山 | Zhong Nanshan | 飞沫 | Droplet Infection | 钟南山 | Zhong Nanshan | 口罩 | Mask |
| 消毒剂 | Sanitizer | 建议 | Suggestion | 疫苗 | Vaccine | 钟南山 | Zhong Nanshan |
| 酒精 | Ethyl alcohol | 风险 | Risk | 武汉 | Wuhan | 患者 | Patient |
| 疫苗 | Vaccine | 酒精 | Ethyl alcohol | 大蒜 | Garlic | 北京 | Beijing |
| 医院 | Hospital | 疾病 | Disease | 大量 | Abundant | 上海 | Shanghai |
| 中国 | China | 证据 | Evidence | 病人 | Patient | 意大利 | Italy |
| 病人 | Patient | 感染者 | Infected person | 日本 | Japan | 病人 | Patient |
| 症状 | Symptom | 人群 | Crowd | 抗体 | Antibody | 疫苗 | Vaccine |
| 医用 | Medical | 儿童 | Children | 院士 | Academician | 病例 | Patient case |
| 风险 | Risk | 居家 | Staying at home | 医生 | Doctor | 湖北 | Hubei province |
| 北京 | Beijing | 通风 | Ventilating | 病毒感染 | Virus infection | 人员 | Staff |
| 人员 | Staff | 人员 | Staff | 空气 | Air | 成都 | Chengdu |
| 病例 | Patient case | 物品 | Goods | 白酒 | Liquor | 院士 | Academician |
| 意大利 | Italy | 效果 | Effect | 防病毒 | Anti-virus | 入境 | Immigration |
| 建议 | Suggestion | 传染性 | Infectiousness | 小时 | Hours | 医生 | Doctor |
| 上海 | Shanghai | 人类 | Human | 病情 | Illness state | 视频 | Video |
| 飞沫 | Droplet Infection | 距离 | Distance | 中国 | China | 全国 | Nationwide |
| 抗体 | Antibody | 核酸检测 | PCR test | 流鼻涕 | Rhinorrhea | 全部 | Entire |
| 院士 | Academician | 疫苗 | Vaccine | 纸尿裤 | Diaper | 阳性 | Positive |
| 感染者 | Infected person | 动物 | Animal | 气溶胶 | Aerosol | 员工 | Staff |
| 阳性 | Positive | 食品 | Food | 二氧化氯 | Chlorine dioxide | 印度 | India |
| 核酸检测 | PCR test | 情况 | Situation | 消毒剂 | Sanitizer | 国家 | Country |
| 医生 | Doctor | 传播者 | Spreader | 牛羊肉 | Beef and mutton | 物资 | Goods |
| 疾病 | Disease | 重症 | Severe case | 喉咙 | Throat | 酒精 | Ethyl alcohol |
| 湖北 | Hubei province | 手部 | Hand | 肥皂 | Soap | 特朗普 | Trump |
| 空气 | Air | 手套 | Glove | 食品 | Food | 风险 | Risk |
| 证据 | Evidence | 传染病 | Infectious disease | 食用 | Edible | 广州 | Canton |
| 人类 | Human | 紫外线 | Ultraviolet ray | 瘟疫 | Plague | 医疗 | Medical treatment |

**Appendix 2:** Translation for Chinese displayed in Figure 3 and Figure 4

| All records | | Records labeled as true | | Records labeled as false | | Records labeled uncertain | |
| --- | --- | --- | --- | --- | --- | --- | --- |
| Chinese | Translation | Chinese | Translation | Chinese | Translation | Chinese | Translation |
| 新冠 | Covid-19 | 新冠 | Covid-19 | 新型 | Novel | 新冠 | Covid-19 |
| 病毒 | Virus | 病毒 | Virus | 预防 | Prevention | 感染 | Infection |
| 冠状病毒 | Coronavirus | 感染 | Infection | 冠状病毒 | Coronavirus | 武汉 | Wuhan |
| 肺炎 | Pneumonia | 肺炎 | Pneumonia | 病毒 | Virus | 肺炎 | Pneumonia |
| 新型 | Novel | 冠状病毒 | Coronavirus | 新冠 | Covid-19 | 病毒 | Virus |
| 感染 | Infection | 时 | Hours, during | 肺炎 | Pneumonia | 月 | Months |
| 口罩 | Mask | 洗手 | Washing hands | 消毒 | Sterilizing | 天 | Days |
| 预防 | Prevention | 没有 | No, without | 感染 | Infection | 美国 | U.S. |
| 没有 | No, without | 消毒剂 | Sanitizer | 口罩 | Mask | 口罩 | Mask |
| 传播 | Spreading | 接触 | Touching | 酒精 | Ethyl alcohol | 成都 | Chengdu |
| 可能 | Maybe | 口罩 | Mask | 治疗 | Curing | 广州 | Guangzhou |
| 使用 | Using | 戴 | Wearing | 有效 | Effective | 高速 | High way |
| 隔离 | Quarantine | 检测 | Test | 30 | 30 | 疫苗 | Vaccine |
| 会 | Able | 患者 | Patient | 秒 | Seconds | 会 | Able |
| 不能 | Unable | 传播 | Spreading | 传播 | Spreading | 日 | Date |
| 接触 | Touching | 可能 | Maybe | 75% | 75% | 病人 | Patient |
| 治疗 | Curing | 含氯 | Chlorinated | 美国 | U.S. | 养老院 | Retirement home |
| 医院 | Hospital | 治疗 | Curing | 武汉 | Wuhan | 消毒 | Sterilizing |
| 防护 | Protection | 有效 | Effective | 疫情 | Epidemic | 中国 | China |
| 疫苗 | Vaccine | 使用 | Using | 喝 | Drinking | 确诊 | Confirming |
| 时 | Hours, during | 疫苗 | Vaccine | 医生 | Doctor | 抗体 | Antibody |
| 有效 | Effective | 疾病 | Disease | 钟南山 | Zhong Nanshan | 日晚 | Evening |
| 避免 | Avoiding | 呼吸道 | Respiratory tract | 度 | Degree | 发现 | Discovering |
| 武汉 | Wuhan | 预防 | Prevention | 容易 | Easy | 上海 | Shanghai |
| 清洁 | Clean | 不能 | Unable | 中国 | China | 隔离 | Quarantine |
| C | C | 新型 | Novel | 喉咙 | Throat | 加拿大 | Canada |
| 佩戴 | Wearing | 应 | Should | 日本 | Japan | 京东 | JD.com |
| 中 | In, China | 中 | In, China | SARS | SARS | 23 | 23 |
| 容易 | Easy | 核酸 | Pcr | 开水 | Boiled water | 增加 | Increasing |
| 人类 | Human | 天 | Days | 戴 | Wearing | 宾馆 | Hotel |
